# The long and winding road of reverse genetics in *Trypanosoma cruzi*

**DOI:** 10.15698/mic2021.09.758

**Published:** 2021-08-05

**Authors:** Miguel A. Chiurillo, Noelia Lander

**Affiliations:** 1 Department of Biological Sciences, University of Cincinnati, Cincinnati, OH 45221, USA.

**Keywords:** Chagas disease, CRISPR/Cas9, gene knockout, gene tagging, gene knock-in, genome editing, trypanosomes

## Abstract

Trypanosomes are early divergent protists with distinctive features among eukaryotic cells. Together with *Trypanosoma brucei* and *Leishmania* spp., *Trypanosoma cruzi* has been one of the most studied members of the group. This protozoan parasite is the causative agent of Chagas disease, a leading cause of heart disease in the Americas, for which there is no vaccine or satisfactory treatment available. Understanding *T. cruzi* biology is crucial to identify alternative targets for antiparasitic interventions. Genetic manipulation of *T. cruzi* has been historically challenging. However, the emergence of CRISPR/Cas9 technology has significantly improved the ability to generate genetically modified *T. cruzi* cell lines. Still, the system alone is not sufficient to answer all biologically relevant questions. In general, current genetic methods have limitations that should be overcome to advance in the study of this peculiar parasite. In this brief historic overview, we highlight the strengths and weaknesses of the molecular strategies that have been developed to genetically modify *T. cruzi*, emphasizing the future directions of the field.

Trypanosomatids (order Kinetoplastidae) belong to an early divergent branch of eukaryotes, the supergroup Discoba, presenting unique characteristics that significantly distinguish them from their vertebrate hosts [1]. The most studied members of this taxonomic group (*Trypanosoma brucei, Trypanosoma cruzi* and *Leishmania* spp.) produce important infectious diseases in humans and cattle. *T. cruzi* is the causative agent of Chagas disease, a neglected tropical disease considered one of the leading causes of heart disease in the world, for which there is no vaccine or satisfactory treatment available. This slow-progressing illness affects about 6-7 million people worldwide and is the most prevalent parasitic disease in the Americas, causing more than 10,000 deaths per year [2]. From the affected population, about 2-3 million people are chronic cases developing serious irreversible heart damage and gastrointestinal or neurological complications [3]. *T. cruzi* is mainly transmitted to humans by contact with feces/urine of infected triatomine bugs. Historically, vector-borne infections were confined to continental rural areas in Latin America but reports of seropositive cases have increased in non-endemic countries of Europe, North America, and the Pacific, mainly as a consequence of global human migrations [4]. But probably the most alarming fact about Chagas disease is that most infected individuals do not know they are sick, remaining undiagnosed until symptoms appear and it is too late to be treated and cured [5]. Currently, there are only two accepted drugs to treat Chagas disease, but they are not FDA-approved for use in adults. Besides their adverse side effects, the efficacy of these drugs decreases the longer a person has been infected. Consequently, the development of new non-toxic chemotherapies to treat chronic patients of Chagas disease is extremely necessary.

Understanding *T. cruzi* biology is critical for the identification of unique structural, metabolic and physiological features that lead to the emergence of new non-toxic and accessible therapies to treat Chagas disease. In this regard, functional analyses of *T. cruzi* essential genes contribute to reveal basic aspects of eukaryotic evolution and biology, as well as to identify and validate new targets for antiparasitic interventions. Genetic manipulation of *T. cruzi* has been historically challenging, as compared to other pathogenic protozoans. However, the adaptation of the prokaryotic CRISPR/Cas9 system for genome editing to *T. cruzi* [6, 7], has significantly improved the ability to generate genetically modified cell lines, becoming a powerful tool for the functional study of proteins in this organism. Still, the system alone is not sufficient to answer all biologically relevant questions. In general, current genetic methods have limitations that should be overcome to advance in the study of this peculiar parasite. In a historic overview of the molecular strategies that have been developed to genetically modify *T. cruzi*, from the generation of the first knockout cell line to the use of CRISPR/Cas9 for genome editing, we can highlight the strengths and weaknesses of this revolutionary molecular tool, emphasizing the predicted directions in the field.

The first *T. cruzi* null mutant was reported in 1993, when the *gp72* gene was ablated using a conventional knockout strategy [8]. Since then and until CRISPR emergence 20 years later, only 20 publications reported attempts to delete *T. cruzi* genes, generating either null mutants (KO) or single-allele deletion mutants (SKO) in this parasite. With the adaptation of CRISPR/Cas9 technology for genome editing in *T. cruzi* [7, 9] this number increased to 67 genes in just six years, during which 35 genes were targeted by CRISPR/Cas9 strategies (**Figure 1**). This data highlights the contribution of CRISPR to reverse genetics in this parasite. In this regard, two aspects have been crucial to advance in the genetic manipulation of *T. cruzi*. The first one is the development of specific expression vectors for this parasite, which established the beginning of reverse genetics in *T. cruzi* in the early ‘90s and allowed the recent implementation of CRISPR technology in this organism. These vectors were designed considering that transcription is polycistronic in *T. cruzi*. Most of them include a strong ribosomal promoter, UTR sequences flanking the gene of interest (necessary for mRNA processing), and DNA sequences that allow integration of the construct into silent loci of the parasite genome [10]. The second aspect is the availability of accurate *T. cruzi* genomic data, which is critical to develop gene editing strategies. The first *T. cruzi* genome sequence corresponding to the hybrid strain CL Brener was released in 2005, after more than a decade of effort involving laboratories and researchers from different countries [11, 12]. Based on this genomic data, four years later a chromosomal-level assembly for each CL Brener haplotype was generated, including bacterial artificial choromosome (BAC) end sequences and synteny information from the *T. brucei* genome. [13]. Since then, new sequencing technologies have been used to assemble the genomes of several *T. cruzi* strains with improved contiguity and assembly accuracy [14–16]. This genomic information has been essential to catalyze reverse genetic approaches for the functional analysis of genes in *T. cruzi*. However, *T. cruzi* genomic data still shows limitations due to the presence of extensive DNA repetitive regions, including large multigene families, and chromosome copy number variations characteristic of this parasite [10, 12]. Therefore, additional and more accurate genomic sequences of different *T. cruzi* strains are necessary to increase the use and efficiency of gene editing techniques in this parasite.

**Figure 1:**
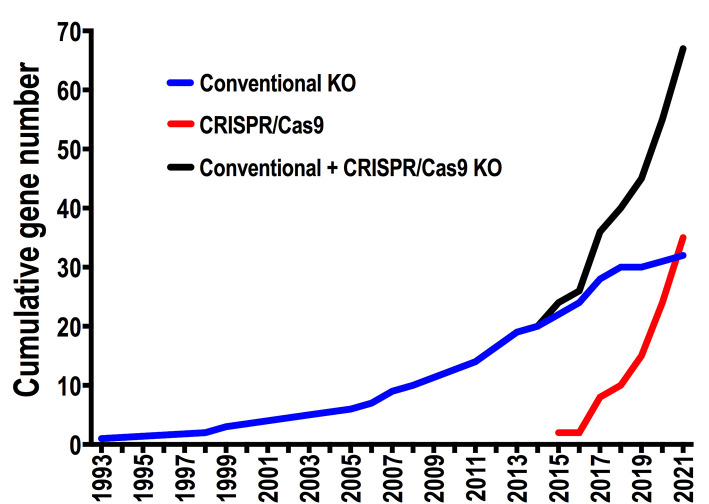
FIGURE 1: Impact of CRISPR/Cas9 technology on the generation of null mutants in *Trypanosoma cruzi*. Linear plots show the cumulative number of endogenous genes for which ablation attempts have been published per year in *T. cruzi*, using conventional knockout (blue), CRISPR/Cas9 (red), and the total number using either of the two methods (black). Only the first attempts reported for each gene were considered in the analysis.

Revisiting recent genetic achievements within trypanosomatids, CRISPR/Cas9 technology was first used to modify the genome of *T. cruzi*. Genome editing was initially adapted to this organism six years ago by constitutive expression of Cas9 and either transient [7] or constitutive expression of the sgRNA [6]. In these studies, different conditions were tested such as targeting individual genes or multigene families, the use of one or two plasmid vectors for the expression of Cas9 and sgRNA, and the efficiency of the system with or without a DNA donor template to promote homology directed repair (HDR). In the absence of DNA donor, double strand break (DSB) repair in *T. cruzi* was carried out by microhomology-mediated end joining (MMEJ) [7]. The inclusion of a DNA donor to induce HDR increased the genome editing efficiency to 100%, generating a homogeneous double knockout population by replacement of both alleles with a single resistance marker in a few weeks, a result never achieved in *T. cruzi* before [6]. This strategy has been successfully used for endogenous tagging of several genes [9] and for the functional characterization of about 20 proteins (reviewed by [17–19]), including important players in calcium signaling and homeostasis, [20–26], which evidences the usefulness of the system to efficiently modify the genome of *T. cruzi*. It is important to mention that knockout cell lines obtained through this strategy have been complemented with either wild type or mutant versions of the added-back gene [21]. In addition, *in vivo* site-directed mutagenesis was used for the first time to study the mitochondrial calcium uniporter subunits *TcMCUb* and *TcMCUc* by CRISPR/Cas9-mediated gene knock-in. In general, research in cell signaling has made significant progress with the use of CRISPR/Cas9 in the generation of calcium-related mutant cell lines in *T. cruzi*, providing new insights into the adaptations of trypanosomes to their particular life styles and supporting the view that one of the main roles of calcium in this parasite is linked to the regulation of intramitochondrial dehydrogenases, with important implications in cell bioenergetics and life/death decisions during *T. cruzi* life cycle [27].

Another approach for genome editing in *T. cruzi* involves the delivery of a ribonucleoprotein (RPN) complex composed by a smaller Cas9 (SaCas9), and *in vitro*-transcribed sgRNAs by nucleofection into different stages of the parasite's life cycle (epimastigotes and trypomastigotes) and different *T. cruzi* strains [28] resulting efficient for the disruption of reporter genes. Although the system did not generate null mutants of endogenous genes, selection-free endogenous tagging was achieved using this method, that can be potentially used for gene editing in other trypanosomatids. Alternatively, a cloning-free CRISPR/Cas9-PCR-based method that involves cell lines constitutively expressing Cas9 and T7 RNA polymerase has been implemented in *T. cruzi* [29]. In this system, a bioluminescent/fluorescent *T. cruzi* cell line is used to follow infection kinetics in a murine model and visualize individual fluorescent parasites in tissue sections. The reporter cell line was also engineered for CRISPR/Cas9 functionality to facilitate genome editing, thus generating bioluminescent/fluorescent/null mutants to evaluate their phenotype *in vivo*. Even though the method requires the insertion of two different resistance markers to replace both alleles of the gene, this promising system should be further explored for drug target validation in *T. cruzi*. So far, the different CRISPR strategies that have been developed in trypanosomatids indicate that gene editing efficiency is much higher in the presence of a DNA donor template than in its absence, confirming that HDR is more efficient that MMEJ for DSB repair in *T. cruzi*. This information is very useful when designing CRISPR experiments in this parasite, albeit the delivery of a DNA donor molecule could restrict high throughput gene ablation studies.

Despite the progress achieved in *T. cruzi* reverse genetics with the different methods developed, 67 ablated genes in 31 years represents a very low number, corresponding to less than 0.5% of the ~12,000 annotated protein-coding genes in *T. cruzi.* The number looks even more negligible when compared with other kinetoplastid species such as *T. brucei* (agent of sleeping sickness)*,* in which almost all protein-coding genes have been analyzed by downregulation or deletion using single gene strategies or high-throughput screenings, mainly based on RNAi technology [30, 31]. Moreover, in some Leishmania spp., which like *T. cruzi*, lack the RNAi machinery and are known to be refractory to genetic manipulation, near 500 genes have been analyzed by gene ablation so far [31–33]. In addition, flagellar and kinome gene deletion libraries analyzing 82 and 204 genes, respectively, have been recently generated in *Leishmania mexicana* (the agent of cutaneous leishmaniasis) using CRISPR/Cas9 [32, 33]. However, no genome-wide functional analyses, or high throughput downregulation studies has been reported in *T. cruzi*.

The availability of reliable and efficient inducible systems is crucial to investigate essential genes. In this regard, loss-of-function analyses of *T. cruzi* essential genes using CRISPR/Cas9 methodologies have limitations. As non-infective epimastigotes are easily cultured and can be genetically modified by current available protocols, most of the genetic interventions in *T. cruzi* have been done in this insect-specific stage. However, it is more relevant to study the essentiality of potential drug targets using the mammalian stages of the parasite's life cycle. *T. cruzi* amastigotes have been directly manipulated by CRISPR/Cas9 [34], however this method has the same constraints to evaluate gene essentiality as those used in epimastigotes with conventional, non-inducible gene editing methods, where a gene is considered essential when it is not possible to obtain viable null mutants. Recently, a CRISPR/Cas9-riboswitch-based method has been developed for downregulation of gene expression in *T. cruzi* [35]. With this strategy, endogenous genes can be tagged with the *glm*S ribozyme from *Bacillus subtilis*, and their expression downregulated at the post-transcriptional level. Although the system is not inducible due to the apparent endogenous production of glucosamine 6-phosphate, it has resulted useful to obtain knockdowns of essential genes in *T. cruzi* [35, 36]. In addition, the CRE-*lox* recombination system has been tested in *T. cruzi* epimastigotes and tissue culture trypomastigotes [37]. Albeit it has not been used to manipulate endogenous genes yet, the adaptation of the CRE-*lox* system to generate an inducible CRISPR/Cas9 knockout strategy could contribute to expand the available toolbox to manipulate this parasite. But so far, using the currently available methodologies, the generation of loss-of-function mutants to target multigene families is practically impossible. Interestingly, a new CRISPR-associated nuclease (Cas13) that targets RNA instead of DNA has been described in prokaryotes [38–41]. The system was successfully adapted to mammalian cells, where Cas13 acts as an RNA-guided RNAse, without modifying the genomic DNA sequence [38, 41]. This new type of Cas nucleases is a promising alternative for the study of essential genes and multigene families, and to achieve large-scale functional screenings in *T. cruzi*.

Long story short, while the successful implementation of the CRISPR/Cas9 system has promoted an important momentum in the genetic manipulation of *T. cruzi*, the field is still missing the development of conditional knockouts or robust systems for inducible knockdown, which would further accelerate the identification and validation of alternative drug targets to treat Chagas disease, as well as the generation of knowledge on the biology of early divergent eukaryotes.

## References

[1] Adl SM, Bass D, Lane CE, Lukes J, Schoch CL, Smirnov A, Agatha S, Berney C, Brown MW, Burki F, Cardenas P, Cepicka I, Chistyakova L, Del Campo J, Dunthorn M, Edvardsen B, Eglit Y, Guillou L, Hampl V, Heiss AA, Hoppenrath M, James TY, Karnkowska A, Karpov S, Kim E, Kolisko M, Kudryavtsev A, Lahr DJG, Lara E, Le Gall L (2019). Revisions to the Classification, Nomenclature, and Diversity of Eukaryotes.. J Eukaryot Microbiol.

[2] Montgomery SP, Starr MC, Cantey PT, Edwards MS, Meymandi SK (2014). Neglected parasitic infections in the United States: Chagas disease.. Am J Trop Med Hyg.

[3] World Health Organization (2021). Chagas disease (also known as American trypanosomiasis).. https://www.who.int/news-room/fact-sheets/detail/chagas-disease-(american-trypanosomiasis).

[4] Forsyth CJ, Hernandez S, Olmedo W, Abuhamidah A, Traina MI, Sanchez DR, Soverow J, Meymandi SK (2016). Safety Profile of Nifurtimox for Treatment of Chagas Disease in the United States.. Clin Infect Dis.

[5] Manne-Goehler J, Reich MR, Wirtz VJ (2015). Access to care for Chagas disease in the United States: a health systems analysis.. Am J Trop Med Hyg.

[6] Lander N, Li ZH, Niyogi S, Docampo R (2015). CRISPR/Cas9-Induced Disruption of Paraflagellar Rod Protein 1 and 2 Genes in Trypanosoma cruzi Reveals Their Role in Flagellar Attachment.. mBio.

[7] Peng D, Kurup SP, Yao PY, Minning TA, Tarleton RL (2014). CRISPR-Cas9-mediated single-gene and gene family disruption in Trypanosoma cruzi.. mBio.

[8] Cooper R, de Jesus AR, Cross GA (1993). Deletion of an immunodominant Trypanosoma cruzi surface glycoprotein disrupts flagellum-cell adhesion.. J Cell Biol.

[9] Lander N, Chiurillo MA, Docampo R (2016). Genome Editing by CRISPR/Cas9: A Game Change in the Genetic Manipulation of Protists.. J Eukaryot Microbiol.

[10] Bartholomeu DC, Teixeira SMR, Cruz AK (2021). Genomics and functional genomics in Leishmania and Trypanosoma cruzi: statuses, challenges and perspectives.. Mem Inst Oswaldo Cruz.

[11] Ramirez JL (2020). Trypanosoma cruzi Genome 15 Years Later: What Has Been Accomplished?. Trop Med Infect Dis.

[12] El-Sayed NM, Myler PJ, Bartholomeu DC, Nilsson D, Aggarwal G, Tran AN, Ghedin E, Worthey EA, Delcher AL, Blandin G, Westenberger SJ, Caler E, Cerqueira GC, Branche C, Haas B, Anupama A, Arner E, Aslund L, Attipoe P, Bontempi E, Bringaud F, Burton P, Cadag E, Campbell DA, Carrington M, Crabtree J, Darban H, da Silveira JF, de Jong P, Edwards K (2005). The genome sequence of Trypanosoma cruzi, etiologic agent of Chagas disease.. Science.

[13] Weatherly DB, Boehlke C, Tarleton RL (2009). Chromosome level assembly of the hybrid Trypanosoma cruzi genome.. BMC Genomics.

[14] Diaz-Viraque F, Pita S, Greif G, de Souza RCM, Iraola G, Robello C (2019). Nanopore Sequencing Significantly Improves Genome Assembly of the Protozoan Parasite Trypanosoma cruzi.. Genome Biol Evol.

[15] Berna L, Rodriguez M, Chiribao ML, Parodi-Talice A, Pita S, Rijo G, Alvarez-Valin F, Robello C (2018). Expanding an expanded genome: long–read sequencing of Trypanosoma cruzi.. Microb Genom.

[16] Wang W, Peng D, Baptista RP, Li Y, Kissinger JC, Tarleton RL (2021). Strain-specific genome evolution in Trypanosoma cruzi, the agent of Chagas disease.. PLoS Pathog.

[17] Lander N, Chiurillo MA (2019). State-of-the-art CRISPR/Cas9 Technology for Genome Editing in Trypanosomatids.. J Eukaryot Microbiol.

[18] Yagoubat A, Corrales RM, Bastien P, Leveque MF, Sterkers Y (2020). Gene Editing in Trypanosomatids: Tips and Tricks in the CRISPR-Cas9 Era.. Trends Parasitol.

[19] Kirti A, Sharma M, Rani K, Bansal A (2021). CRISPRing protozoan parasites to better understand the biology of diseases.. Prog Mol Biol Transl Sci.

[20] Chiurillo MA, Lander N, Bertolini MS, Storey M, Vercesi AE, Docampo R (2017). Different Roles of Mitochondrial Calcium Uniporter Complex Subunits in Growth and Infectivity of Trypanosoma cruzi.. MBio.

[21] Chiurillo MA, Lander N, Bertolini MS, Vercesi AE, Docampo R (2019). Functional analysis and importance for host cell infection of the Ca(2+)-conducting subunits of the mitochondrial calcium uniporter of Trypanosoma cruzi.. Mol Biol Cell.

[22] Bertolini MS, Chiurillo MA, Lander N, Vercesi AE, Docampo R (2019). MICU1 and MICU2 Play an Essential Role in Mitochondrial Ca(2+) Uptake, Growth, and Infectivity of the Human Pathogen Trypanosoma cruzi.. MBio.

[23] Lander N, Chiurillo MA, Bertolini MS, Storey M, Vercesi AE, Docampo R (2018). Calcium-sensitive pyruvate dehydrogenase phosphatase is required for energy metabolism, growth, differentiation, and infectivity of Trypanosoma cruzi.. J Biol Chem.

[24] Chiurillo MA, Lander N, Vercesi AE, Docampo R (2020). IP3 receptor-mediated Ca(2+) release from acidocalcisomes regulates mitochondrial bioenergetics and prevents autophagy in Trypanosoma cruzi.. Cell Calcium.

[25] Negreiros RS, Lander N, Chiurillo MA, Vercesi AE, Docampo R (2021). Mitochondrial Pyruvate Carrier Subunits Are Essential for Pyruvate-Driven Respiration, Infectivity, and Intracellular Replication of Trypanosoma cruzi.. mBio.

[26] Dos Santos GRR, Rezende Leite AC, Lander N, Chiurillo MA, Vercesi AE, Docampo R (2021). Trypanosoma cruzi Letm1 is involved in mitochondrial Ca(2+) transport, and is essential for replication, differentiation, and host cell invasion.. FASEB J.

[27] Docampo RV, A. E., Huang, Lander, Chiurillo, Bertolini (2021). Mitochondrial Ca2+ homeostasis in trypanosomes.. Int Rev Cell Mol Biol.

[28] Soares Medeiros LC, South L, Peng D, Bustamante JM, Wang W, Bunkofske M, Perumal N, Sanchez-Valdez F, Tarleton RL (2017). Rapid, Selection-Free, High-Efficiency Genome Editing in Protozoan Parasites Using CRISPR-Cas9 Ribonucleoproteins.. MBio.

[29] Costa FC, Francisco AF, Jayawardhana S, Calderano SG, Lewis MD, Olmo F, Beneke T, Gluenz E, Sunter J, Dean S, Kelly JM, Taylor MC (2018). Expanding the toolbox for Trypanosoma cruzi: A parasite line incorporating a bioluminescence-fluorescence dual reporter and streamlined CRISPR/Cas9 functionality for rapid in vivo localisation and phenotyping.. PLoS Negl Trop Dis.

[30] Alsford S, Turner DJ, Obado SO, Sanchez-Flores A, Glover L, Berriman M, Hertz-Fowler C, Horn D (2011). High-throughput phenotyping using parallel sequencing of RNA interference targets in the African trypanosome.. Genome Res.

[31] Jones NG, Catta-Preta CMC, Lima A, Mottram JC (2018). Genetically Validated Drug Targets in Leishmania: Current Knowledge and Future Prospects.. ACS Infect Dis.

[32] Baker N, Catta-Preta CMC, Neish R, Sadlova J, Powell B, Alves-Ferreira EVC, Geoghegan V, Carnielli JBT, Newling K, Hughes C, Vojtkova B, Anand J, Mihut A, Walrad PB, Wilson LG, Pitchford JW, Volf P, Mottram JC (2021). Systematic functional analysis of Leishmania protein kinases identifies regulators of differentiation or survival.. Nat Commun.

[33] Beneke T, Demay F, Hookway E, Ashman N, Jeffery H, Smith J, Valli J, Becvar T, Myskova J, Lestinova T, Shafiq S, Sadlova J, Volf P, Wheeler RJ, Gluenz E (2019). Genetic dissection of a Leishmania flagellar proteome demonstrates requirement for directional motility in sand fly infections.. PLoS Pathog.

[34] Takagi Y, Akutsu Y, Doi M, Furukawa K (2019). Utilization of proliferable extracellular amastigotes for transient gene expression, drug sensitivity assay, and CRISPR/Cas9-mediated gene knockout in Trypanosoma cruzi.. PLoS Negl Trop Dis.

[35] Lander N, Cruz-Bustos T, Docampo R (2020). A CRISPR/Cas9-riboswitch-Based Method for Downregulation of Gene Expression in Trypanosoma cruzi.. Front Cell Infect Microbiol.

[36] Mantilla BS, Amaral LDD, Jessen HJ, Docampo R (2021). The Inositol Pyrophosphate Biosynthetic Pathway of Trypanosoma cruzi.. ACS Chem Biol.

[37] Pacheco-Lugo LA, Saenz-Garcia JL, Diaz-Olmos Y, Netto-Costa R, Brant RSC, DaRocha WD (2020). CREditing: a tool for gene tuning in Trypanosoma cruzi.. Int J Parasitol.

[38] Abudayyeh OO, Gootenberg JS, Essletzbichler P, Han S, Joung J, Belanto JJ, Verdine V, Cox DBT, Kellner MJ, Regev A, Lander ES, Voytas DF, Ting AY, Zhang F (2017). RNA targeting with CRISPR-Cas13.. Nature.

[39] Cox DBT, Gootenberg JS, Abudayyeh OO, Franklin B, Kellner MJ, Joung J, Zhang F (2017). RNA editing with CRISPR-Cas13.. Science.

[40] East-Seletsky A, O'Connell MR, Burstein D, Knott GJ, Doudna JA (2017). RNA Targeting by Functionally Orthogonal Type VI-A CRISPR-Cas Enzymes.. Mol Cell.

[41] Konermann S, Lotfy P, Brideau NJ, Oki J, Shokhirev MN, Hsu PD (2018). Transcriptome Engineering with RNA-Targeting Type VI-D CRISPR Effectors.. Cell.

